# Imported Congenital Rubella Syndrome, United States, 2017

**DOI:** 10.3201/eid2404.171540

**Published:** 2018-04

**Authors:** Roukaya Al Hammoud, James R. Murphy, Norma Pérez

**Affiliations:** The University of Texas Health Science Center, Houston, Texas, USA

**Keywords:** Congenital, rubella, pregnant, fever, rash, arthralgia, leucocoria, pulmonary stenosis, cataract, newborn, cardiac deceleration, systolic heart murmur, neonatal reflexes, diffuse coarse trabecular pattern, striated appearance of the metaphysis, lucent linear areas, Saudi Arabia, United States, viruses

## Abstract

Although transmission of rubella virus within the United States is rare, the risk for imported cases persists. We describe a rubella case in a newborn, conceived in Saudi Arabia, in Texas during 2017, highlighting the importance of active surveillance and early diagnosis of this disease.

A full-term male infant was born in Houston, Texas, USA, in early 2017 to a 29-year-old woman from Pakistan; this pregnancy was her first. Delivery was by emergent cesarean section because of fetal cardiac decelerations. The mother had lived in Saudi Arabia for 3 years before traveling to the United States in her third trimester of pregnancy. Early in pregnancy, while in Saudi Arabia, she had acute onset of fever and rash, then arthralgia. Symptoms resolved within a week without medical treatment. She reported prenatal care in Saudi Arabia but had no records with her; she knew of no ill contacts during pregnancy. At delivery, she had negative results for HIV and negative rapid plasma reagin but positive rubella IgG titers (>500 IU/Ml; reference, positive >10 IU/mL). 

The infant was transferred to The University of Texas Health Science Center (Houston, Texas, USA). Birthweight and head circumference were below the third percentile. Symptoms were respiratory distress, left leucocoria (Figure), systolic heart murmur, and depressed neonatal reflexes. Laboratory evaluation showed normal peripheral leukocyte count, hemoglobin, and liver enzymes and platelet count of 93,000/mm^3^. Because of suspected congenital rubella infection, we placed the patient on contact isolation. Tests for cytomegalovirus and toxoplasma were negative. We considered congenital Zika syndrome, but no testing was done. An ophthalmologic exam confirmed left cataract without retinal involvement. Chest radiograph showed clear lungs; echocardiogram showed supravalvular pulmonary stenosis and patent ductus arteriosus. Cerebrospinal spinal fluid (CSF) analysis showed normal leukocyte, glucose, and protein levels. Blood and CSF cultures were negative. On the fourth day of life, blood rubella IgG was >500 IU/mL (reference, immune ≥10 IU/mL), and blood rubella IgM was >400 AU/mL (reference range 20–24.9 AU/mL). Ultrasound examination of the brain was unremarkable. Radiographic evaluation of long bones showed diffuse coarse trabecular pattern, striated appearance of the metaphysis, and lucent linear areas. Audiometry brainstem response testing failed in the left ear. Thrombocytopenia self-resolved. 

**Figure:**
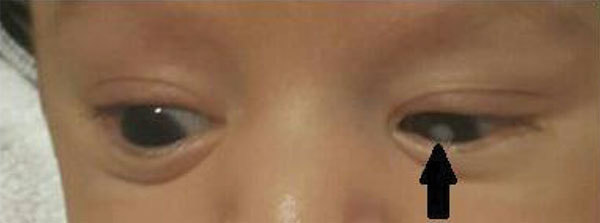
Left eye cataract (arrow) in case-patient with congenital rubella syndrome, Texas, USA, 2017. Patient was 4 weeks of age.

We reported the case to the local health department. We sent no clinical specimens for rubella virus detection. The patient was discharged on his tenth day of life and had uncomplicated pulmonary valvuloplasty and cataract removal surgery by 6 weeks of age. The infectious disease team last saw the patient at 2 months of age; at that time, he was developing well, but his growth was borderline. The patient and his family traveled to Pakistan 3 months after birth.

Rubella is of global public health concern; >100,000 cases of congenital rubella syndrome (CRS) are reported annually worldwide (*1*). An immunization program resulted in rubella elimination in the United States during 2004 (*2*). Currently, the Centers for Diseases Control and Prevention (CDC) estimates that <10 persons are reported to have rubella annually in the United States (*2*). During the 8 years after rubella was eliminated (2004–2012), 79 of rubella cases were reported, including in case-patients with no travel history (*3*). For the same period, 6 CRS cases were reported to CDC, 5 of which were likely imported (*3*). The sixth case was the infant of a US-born vaccinated mother without known risk factors (*4*). During the next 4 years (2013–2016), 5 confirmed CRS cases were reported to CDC, indicating a relative increase in the total number of new cases in the United States. The 5 cases were reported by three states, Illinois (2 cases), New York (2 cases), and Maryland (1 case); infections were likely acquired in Algeria, Pakistan, Yemen, and Nigeria (US Centers for Disease Control and Prevention, 2017 March, pers. comm) (*5*).

During early pregnancy, the mother of the case-patient likely acquired acute rubella infection in Saudi Arabia, which increased its rubella vaccine program in July 2017 to meet control needs (*6*). Maternal immunization records and rubella titers were not available. The infant had positive rubella IgM, cataract, congenital heart disease, microcephaly, unilateral hearing loss, and radiolucent bone disease, meeting criteria for CRS. Screening for rubella titers in early pregnancy is standard in the United States. The presence of positive maternal rubella serology at delivery does not always reflect maternal immunization but can be the result of a rubella infection in early pregnancy. A similar scenario was misleading in a case that was recently reported and resulted in late diagnosis of CRS and subsequent multiple exposures (*6*). 

Rubella-like illness in early pregnancy warrants testing for acute rubella infection, which offers parents an opportunity to decide about pregnancy outcome. For confirmed cases, maternal counseling and pregnancy termination may be considered. Testing for CRS is critical for early disease confirmation, implementation of appropriate infection control, timely reporting, and possible epidemiologic investigation. Infants with CRS shed large quantities of virus from bodily secretions for up to 1 year and can transmit rubella virus to susceptible persons (*7**)*. The presence of unimmunized persons in the United States (for age, personal, or medical reasons) and entry of persons from rubella-endemic countries enable potential circulation of the virus. Despite rubella elimination in the United States, the presence of birth defects compatible with CRS warrants consideration of rubella in addition to other congenital infections.
